# TNF**α** and IL-6 Responses to Particulate Matter *in Vitro*: Variation According to PM Size, Season, and Polycyclic Aromatic Hydrocarbon and Soil Content

**DOI:** 10.1289/ehp.1409287

**Published:** 2015-09-15

**Authors:** Natalia Manzano-León, Jesús Serrano-Lomelin, Brisa N. Sánchez, Raúl Quintana-Belmares, Elizabeth Vega, Inés Vázquez-López, Leonora Rojas-Bracho, Maria Tania López-Villegas, Felipe Vadillo-Ortega, Andrea De Vizcaya-Ruiz, Irma Rosas Perez, Marie S. O’Neill, Alvaro R. Osornio-Vargas

**Affiliations:** 1 Departamento de Investigación Básica, Instituto Nacional de Cancerología, México, DF, México; 2 School of Public Health, University of Alberta, Edmonton, Alberta, Canada; 3 Department of Environmental Health Sciences, and; 4 Department of Epidemiology, School of Public Health, University of Michigan, Ann Arbor, Michigan, USA; 5 Dirección de Investigación y Posgrado, Instituto Mexicano del Petróleo, México, DF, México; 6 Gerencia de Ciencias Ambientales, Instituto Nacional de Investigaciones Nucleares, La Marquesa, Ocoyoacac, Estado de México, México; 7 Instituto Nacional de Ecología y Cambio Climático, México, DF, México; 8 National Autonomous University of Mexico (UNAM) at the National Institute of Genomic Medicine, México, DF, México; 9 Departamento de Toxicología, Cinvestav, México, DF, México; 10 Centro de Ciencias de la Atmósfera, UNAM, México, DF, México; 11 Department of Paediatrics, University of Alberta, Edmonton, Alberta, Canada

## Abstract

**Background:**

Observed seasonal differences in particulate matter (PM) associations with human health may be due to their composition and to toxicity-related seasonal interactions.

**Objectives:**

We assessed seasonality in PM composition and in vitro PM pro-inflammatory potential using multiple PM samples.

**Methods:**

We collected 90 weekly PM10 and PM2.5 samples during the rainy-warm and dry-cold seasons in five urban areas with different pollution sources. The elements, polycyclic aromatic hydrocarbons (PAHs), and endotoxins identified in the samples were subjected to principal component analysis (PCA). We tested the potential of the PM to induce tumor necrosis factor alpha (TNFα) and interleukin 6 (IL-6) secretion in cultured human monocytes (THP-1), and we modeled pro-inflammatory responses using the component scores.

**Results:**

PM composition varied by size and by season. PCA identified two main components that varied by season. Combustion-related constituents (e.g., vanadium, benzo[a]pyrene, benzo[a]anthracene) mainly comprised component 1 (C1). Soil-related constituents (e.g., endotoxins, silicon, aluminum) mainly comprised component 2 (C2). PM from the rainy-warm season was high in C2. PM (particularly PM2.5) from the dry-cold season was rich in C1. Elevated levels of cytokine production were associated with PM10 and C2 (rainy-warm season), whereas reduced levels of cytokine production were associated with PM2.5 and C1 (dry-cold season). TNFα secretion was increased following exposure to PM with high (vs. low) C2 content, but TNFα secretion in response to PM was decreased following exposure to samples containing ≥ 0.1% of C1-related PAHs, regardless of C2 content. The results of the IL-6 assays suggested more complex interactions between PM components and particle size.

**Conclusions:**

Variations in PM soil and PAH content underlie seasonal and PM size–related patterns in TNFα secretion. These results suggest that the mixture of components in PM explains some seasonal differences in associations between health outcomes and PM in epidemiologic studies.

**Citation:**

Manzano-León N, Serrano-Lomelin J, Sánchez BN, Quintana-Belmares R, Vega E, Vázquez-López I, Rojas-Bracho L, López-Villegas MT, Vadillo-Ortega F, De Vizcaya-Ruiz A, Rosas Perez I, O’Neill MS, Osornio-Vargas AR. 2016. TNFα and IL-6 responses to particulate matter in vitro: variation according to PM size, season, and polycyclic aromatic hydrocarbon and soil content. Environ Health Perspect 124:406–412; http://dx.doi.org/10.1289/ehp.1409287

## Introduction

Exposure to particulate matter (PM) has been associated with cardiorespiratory diseases and adverse birth outcomes, among other end points (Pope and Dockery 2006; Shah and Balkhair 2011; Zanobetti et al. 2014). PM is a complex mixture of several components including carbon, metals, organic compounds, ions, and biological elements (Pope and Dockery 2006). Seasonal variability in the chemical composition of PM has been reported for *a*) the content of organic carbon, nitrates, and sulfates in samples collected during 5 years in 187 counties in the United States (Bell et al. 2007); *b*) endotoxins in studies performed in Turin, Italy (Traversi et al. 2010); *c*) nickel (Ni), copper (Cu), zinc (Zn), and lead (Pb) in Venice, Italy (Toscano et al. 2011); and *d*) coarse PM enrichment in water-soluble organic carbon and nitrate during the summer in Los Angeles, California (Cheung et al. 2011).

Evidence also links seasonal changes in PM levels and chemical composition with health outcomes. Associations between carbon monoxide (CO), sulfur dioxide (SO_2_), and PM levels and increased mortality differed according to season in two metropolitan areas in the United States (Moolgavkar 2003). Mortality was higher in association with specific characteristics of PM in Arizona during the spring and summer months (Smith et al. 2000), and similar seasonal increments in summer PM-related mortality were observed in cities in the northeastern United States (Peng et al. 2005).

Simultaneous evaluation of the chemical composition of PM collected in different seasons may help to elucidate the relationship between specific constituents of PM and biological effects. Becker et al. (2005) reported that PM samples collected in North Carolina in the autumn were richer in iron (Fe), silicon (Si), and chromium (Cr) and had greater pro-inflammatory potential than PM samples collected in other seasons. Another study showed a relationship between elevated levels of PM_10_ (particulate matter with mean aerodynamic diameter ≤ 10 μm) with a high content of Saharan desert dust and increased mortality, usually between spring and autumn (Díaz et al. 2012). Mugica et al. (2010) found that seasonal variation in the PM_10_ content of polycyclic aromatic hydrocarbons (PAHs) in Mexico City was associated with differences in toxicity.

We analyzed the season-specific composition and *in vitro* pro-inflammatory potential of PM_10_ and PM_2.5_ (particulate matter with mean aerodynamic diameter ≤ 2.5 μm), using samples collected in the rainy-warm (summer) and dry-cold (winter) seasons from five areas of Mexico City with different urban activities. We assessed the variability in the composition of and in the cell responses to 90 samples by applying multivariate analysis and regression modeling, an approach that is not conventionally used in toxicological studies. This work is part of an ongoing epidemiological study of exposure of pregnant women to air pollution and birth outcomes in Mexico City (O’Neill et al. 2013).

## Methods

*PM sampling.* High-volume air samplers and nitrocellulose membranes (Manzano-León et al. 2013) were used to collect PM_10_ and PM_2.5_ at five sites in Mexico City. Samples were collected weekly in May–August 2009 and November 2010–March 2011, corresponding to the rainy-warm and dry-cold seasons of the year, respectively. Sampling sites were selected on the basis of their proximity to the official sites of Mexico City’s air monitoring network and were located in areas mainly representing industrial, business, and residential activities. The selected residential areas vary in traffic-related pollution, demographics, and urban infrastructure [Instituto Nacional de Ecologia-Secretaría de Medio Ambiente y Recursos Naturales (INE-SEMARNAT) 2011].

PM was mechanically recovered from the membranes (Alfaro-Moreno et al. 2009). Weekly PM samples were pooled by month, site, and size, resulting in 40 samples from the rainy-warm season and 50 from the dry-cold season. We determined the chemical composition (elements, PAHs, and endotoxins) and pro-inflammatory potential [tumor necrosis factor alpha (TNFα) and interleukin 6 (IL-6) production] of each sample.

*Chemical analysis.* Elements. Inductively coupled plasma mass spectrometry (ICP-MS; Agilent 7500a) was used to identify and quantify 33 elements. PM samples (~ 1 mg) were dissolved in 3 mL of deionized water (18.2 MΩ/cm). Before analysis, samples were filtered using nylon membranes (0.2 μm; Millipore GNWP). The analysis was conducted using a flow rate of 1.0 L/min of argon, incident power of 1.39 kW, radiofrequency of 1.76 V, and a discriminator threshold of 9.5 mV. Elements with atomic numbers from 3–82 [lithium (Li) to lead (Pb)] were subjected to three scans of 100 passes each, using 32 channels. Interference equations were used for corrections in all samples (see Vega et al. 2011 for details).

Polycyclic aromatic hydrocarbons (PAHs). Sixteen PAHs that have been commonly assessed in other studies (Camatini et al. 2012; Dergham et al. 2012) were identified by performing high-performance liquid chromatography (HPLC; Agilent HP, 1100 series) using a Nucleosil column (Macherey-Nagel, 265 mm, 100-5C18PAH) with an automatic sample injector and a fluorescence detector. PM samples (~ 1 mg) were extracted with 30 mL of dichloromethane in a microwave oven (CEM, model MarsX). The extracts were concentrated to a volume of 1.0 mL under a gentle stream of ultrapure nitrogen supplied by a nitroevaporator. The extracts were then added to 4.0 mL of acetonitrile and reconcentrated to a volume of 0.5 mL under ultrapure nitrogen. The validation method parameters were linearity (*R* ^2^ > 0.98), accuracy/precision [relative standard deviation (RSD) < 3%], and detection limit (0.004 μg/mL). Quantification limits and percent recovery, depending on the compound, were 0.01–0.03 μg/mL and 60.2–94.2%, respectively.

Endotoxins. Endotoxins were extracted from PM samples (1 mg/mL) using 50 mM Tris buffer (Lonza); the samples were sonicated for 1 hr at 22°C and vortexed every minute during 15 min. Serial dilutions (1:5) were prepared using 500-μL aliquots from each extract to identify an optimal dilution. All samples were handled in glass tubes and plastic materials free of endotoxins. The endotoxin analysis was performed using a kit for the kinetic chromogenic Limulus amebocyte lysate (LAL) assay with a Kinetic-QCL reader and software (Lonza Kinetic-QCL) according to the manufacturer’s instructions. The samples were analyzed in duplicate in 96-well microplates. The endotoxin concentration was determined using *Escherichia coli* O55:B5 endotoxin as a standard (10 endotoxin units per nanogram), and the endotoxin content was expressed as nanograms per milligram of particles. The reported endotoxin content represents the overall activity of various types of endotoxin potentially present in the samples.

*Pro-inflammatory cytokines (TNF*α *and IL-6).* We selected TNFα and IL-6 as the cytokines of interest owing to their known participation as acute response factors in PM-mediated pro-inflammatory responses (Hiraiwa and van Eeden 2013). To evaluate *in vitro* cytokine responses, we used THP-1 cells (a human monocytic cell line) obtained from American Type Culture Collection. The cells were cultured in 10% fetal bovine serum–RPMI (catalog no. A10491, Sigma) containing penicillin (50 U/mL) and streptomycin (50 μg/mL). Cultures were kept at 37°C in a 5% CO_2_/95% air atmosphere.

TNFα and IL-6 were measured in the supernatants of 550,000 cells/mL that were exposed to 80 μg/mL of PM_10_ or PM_2.5_ for 24 hr. All exposures were conducted in 24-well plates maintaining an equivalency of 80 μg/mL to 40 μg/cm^2^. We designed the study considering a fixed PM mass concentration in order to allow the concentrations of the PM constituents to guide the concentration-dependent responses. We anticipated that concentrations of the PM constituents would vary according to size and according to the site at which and season when the PM was collected. In previous work, 80 μg/mL was identified to be the optimal concentration of PM to induce cytokine production with minimal loss of cell viability (up to 10%) (Manzano-León et al. 2013). Cells were kept in serum-free media 24 hr before exposure to PM. Fresh aliquots of PM (1 mg/mL) were prepared, vortexed, and sonicated immediately before each exposure. Subsequently, ELISA (Invitrogen) was performed to determine the levels of TNFα and IL-6 in the supernatants. We conducted three independent experiments with each PM sample. Each experiment represented the average concentration of duplicates. Nonexposed cells were used as negative controls, and their basal cytokine levels (TNFα = 3.225 pg/mL and mean IL-6 = 1.135 pg/mL) were subtracted from the experimental values. Cells exposed to 10 μg/mL lipopolysaccharide (LPS) served as positive controls. The results are expressed in picograms per milliliter.

*Statistical analysis.* The analysis included only constituents that were identified in all samples. Medians, means, and 95% confidence intervals (CIs) were calculated for the PM_10_ and PM_2.5_ constituents and groups of constituents (endotoxins, PAHs, and elements). Seasonal medians were compared using the Mann–Whitney test. Medians, means, and percentages for each PM_10_ and PM_2.5_ constituent were calculated, and medians were compared between seasons using the Mann–Whitney test. All constituents were natural log (ln)–transformed, and principal component analysis (PCA) was used to group the multiple PM constituents into components according to the correlations among constituents. The scree plot criterion was used to choose the number of components to retain (Afifi et al. 2004). Component scores were computed and compared between seasons according to PM size using analysis of variance (ANOVA) and were plotted using different symbols to visualize seasonal patterns of PM composition by PM size.

Similarly, we obtained medians, means, and 95% CIs for TNFα and IL-6 levels by season and by PM size.

Associations between the principal components and the ln(cytokines) were calculated using partial correlation coefficients (*r*) and controlling for PM size. The relative participation of the PAHs in C_1_ (C_1_-related PAHs) by PM size and season was calculated as the percentage of the total content of the constituents grouped in C_1_ and C_2_ (C_1_ + C_2_ content). Regression models adjusting for PM size and C_1_ + C_2_ content were used to evaluate the relationship between the percentage of C_1_-related PAHs and ln(cytokine production). C_1_ + C_2_ content was categorized in quartiles: low; low-medium; medium-high; and high. All statistical analyses were performed using SPSS (v20; IBM) and Stata (v10; StataCorp LP).

## Results

*Seasonal characteristics.* The dry-cold season had the following five-site averages: temperature, 14.8 ± 0.98°C; relative humidity, 39.42 ± 2.92%; accumulated precipitation, 22 ± 5 mm; 1-hr maximum ozone, 0.073 ± 0.026 ppm; PM_10_, 67.8 ± 14.02 μg/m^3^; and PM_2.5_, 31.2 ± 2.59 μg/m^3^. For the rainy-warm season, these values were as follows: temperature, 18.6 ± 0.89°C; relative humidity, 57 ± 2%; accumulated precipitation, 660 ± 78 mm; 1-hr maximum ozone, 0.086 ± 0.027 ppm; PM_10_, 42.6 ± 7.09 μg/m^3^; and PM_2.5_, 21 ± 2 μg/m^3^ [Secretaría del Medio Ambiente, Gobierno del Distrito Federal (SEDEMA) 2015].

*PM chemical composition.* Overall, 32 PM constituents (22 elements, 9 PAHs, and endotoxins) were found in all samples (see Supplemental Material, Tables S1 and S2) and are summarized in Table 1. Constituents not represented in all samples and excluded from the analysis are in Supplemental Material, Table S3. The percentage of the total PM mass consisting of the measured constituents was 6.8% (68.45 μg/mg) PM_10_ in the dry-cold season; 12.6% (126.25 μg/mg) PM_10_ in the rainy-warm season; 4.7% (46.60 μg/mg) PM_2.5_ in the dry-cold season; and 2.8% (27.69 μg/mg) PM_2.5_ in the rainy-warm season.

**Table 1:** Summary statistics for PM constituents according to type of constituent (endotoxins, PAHs, or elements), season, and PM size.

Constituent^*a*^	PM_10_		PM_2.5_	
	Median	Mean (95% CI)	Median	Mean (95% CI)
Endotoxins
Dry-cold	4.6	5.2 (4.5, 5.8)	2.1*	2.3 (1.6, 3.0)
Rainy-warm	4.1	6.6 (4.1, 9.2)	0.5*	1.0 (0.4, 1.5)
PAHs
Dry-cold	47.9	53.4 (42.2, 64.7)	56.7*	71.1 (57.3, 84.8)
Rainy-warm	41.7	37.1 (31.4, 42.8)	24.2*	30.7 (23.0, 38.5)
Elements
Dry-cold	71183.4*	68391.6 (64,978, 71,805)	45683.7*	46529.1 (39870, 53,188)
Rainy-warm	119909.3*	126206.4 (105,260, 147,153)	20376.5*	27657.9 (17820, 37,496)
CI, Confidence interval; PM, particulate matter; PAH, polycyclic aromatic hydrocarbon. ^***a***^Nanograms per milligram PM. *Indicates significant differences between seasons by comparing same PM-size constituents (*p* < 0.05; Mann–Whitney test).				

Individual PM constituents varied by season and by PM size (see Supplemental Material, Tables S1 and S2). On average, the concentrations of the elemental constituents in PM_10_ were higher in the rainy-warm season than in the dry-cold season (126,206 ng/mg vs. 68,391 ng/mg, respectively). Calcium (Ca), sulfur (S), potassium (K), sodium (Na), magnesium (Mg), Si, Fe, aluminum (Al), and zinc (Zn) accounted for 98% of the total elemental concentration in PM_10_ from both seasons, and the concentrations of seven of these elements (Ca, S, K, Na, Si, Fe, and Al) were significantly different between seasons (*p* < 0.05) (see Supplemental Material, Table S1). Median concentrations of PAHs in PM_10_ were slightly higher in the dry-cold season (*p* = 0.052) (Table 1). Average PM_10_ endotoxin concentrations were no different between seasons (*p* = 0.584).

The average concentrations of the elemental constituents in PM_2.5_ were higher in the dry-cold season than in the rainy-warm season (46,529 ng/mg vs. 27,657 ng/mg, respectively). The contents of S, Ca, K, Na, Zn, Mg, Fe, copper (Cu), vanadium (V), Al, and Si accounted for 98% of the elemental concentration in PM_2.5_ samples from the dry-cold season, and Ca, S, Na, K, Zn, Mg, Si, and Cu accounted for 98% of the elemental concentration in PM_2.5_ samples from the rainy-warm season (see Supplemental Material, Table S2). Median concentrations of PAHs in PM_2.5_ samples were higher in the dry-cold season than in the rainy-warm season (56.7 ng/mg vs. 24.2 ng/mg, respectively; *p* = 0.001) (Table 1). Endotoxins were significantly higher in PM_2.5_ from the dry-cold season than in PM_2.5_ from the rainy-warm season (2.1 ng/mg vs. 0.5 ng/mg, respectively; *p* = 0.001).

PM elements dominated the mass of the measured PM constituents, comprising ≤ 99.97% of that fraction. Although the percentage of the PM mass consisting of PAHs was < 1%, the mass of PAHs was nearly three times greater in the dry-cold season than in the rainy-warm season when combining both PM sizes (0.11% vs. 0.04%, respectively). Generally, endotoxin content was higher in PM_10_ than in PM_2.5_, but only PM_2.5_ showed seasonal differences (*p* = 0.001) (Table 1). During the rainy-warm season, the mean concentration of all measured constituents was 4.6 times higher in PM_10_ than in PM_2.5_ (126,250 ng/mg vs. 27,690 ng/mg, respectively), whereas in the dry-cold season, the mean concentration of all measured constituents was 1.5 times higher in PM_10_ than in PM_2.5_ (68,450 ng/mg vs. 46,602 ng/mg, respectively) (see Supplemental Material, Tables S1 and S2).

*Principal component analysis.* PCA was performed and included endotoxins, PAHs (acenaphthylene, benzo[*a*]anthracene, benzo[*a*]pyrene, benzo[*b*]fluoranthene, benzo[*ghi*]perylene, benzo[*k*]fluoranthene, chrysene, fluoranthene, and pyrene), and elements (S, Ca, K, Na, Zn, Mg, Fe, Cu, V, Al, and Si) that accounted for 98% of the concentration of all elements, thus lowering the number of explanatory variables while maximizing the case/variable ratio (*n* >> number of variables) (Legendre and Legendre 2012).

Three principal components were extracted, explaining 74.3% of the total variance (see Supplemental Material, Table S4). Component 1 (C1) explained 43.4% of the total variance and was mainly composed of (in descending order of loading) K, V, S, benzo[*a*]pyrene, benzo[*a*]anthracene, fluoranthene, chrysene, benzo[*k*]fluoranthene, benzo[*ghi*]perylene, Fe, Zn, and benzo[*b*]fluoranthene. Component 2 (C2) accounted for 21.5% of the total variance and was mainly composed of (in descending order of loading) Ca, Mg, endotoxins, Si, Al, and Na. Finally, component 3 (C3) mainly consisted of (in descending order of loading) pyrene and acenaphthylene and explained 9.4% of the total variance.

Seasonal differences of component scores were estimated according to PM size. PM_10_ samples from the rainy-warm season had higher levels of C_2_ and C_3_ (*p* < 0.05) and lower levels of C_1_ (*p* < 0.05) than the samples from the dry-cold season. PM_2.5_ samples showed significant seasonal differences exclusively for C_1_, levels of which were higher in the dry-cold season (*p* < < 0.05) (see Supplemental Material, Figure S1).

**Figure 1:**
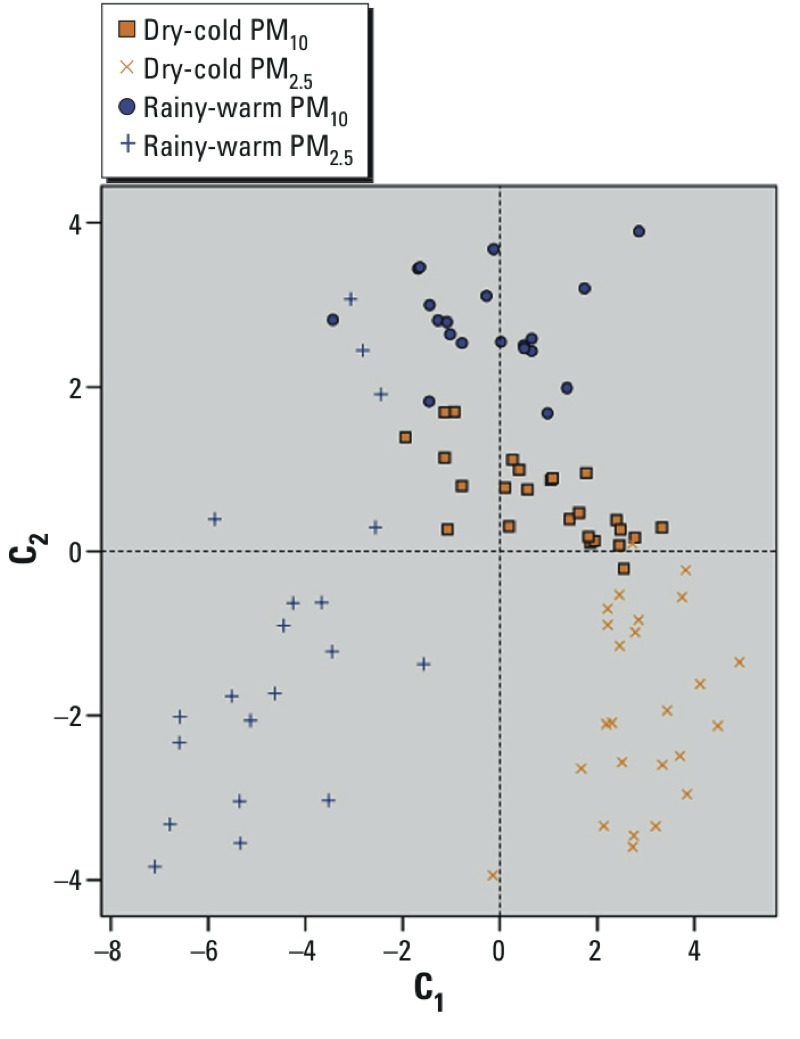
C_1_, C_2_ component scores plot according to PM size and season. Samples (*n* = 90) according to season and PM size (groups) are well differentiated in their content of C_1_ and C_2_. Rainy-warm PM_10_ had high concentrations of C_2_ and moderate concentrations of C_1_; dry-cold PM_10_ presented moderate C_1_ concentrations and moderate-high concentrations of C_2_; rainy-warm PM_2.5_ generally had low concentrations of C_1_ and C_2_; dry-cold PM_2.5_ showed high concentrations of C_1_ and low concentrations of C_2_. The zeroes on both axes correspond to the mean of all samples, and integer values are based on the component loading of the observation and the standardized value of the original variable, summed over all variables.

There were four distinct patterns of distribution of C_1_ and C_2_ by size and season in the 90 PM samples: *a*) PM_2.5_ from the rainy-warm season had low C_1_ and C_2_ content; *b*) PM_10_ from the dry-cold season had approximately average C_1_ content and just above average C_2_ content; *c*) PM_10_ from the rainy-warm season had average C_1_ content and high C_2_ content; and *d*) PM_2.5_ from the dry-cold season had low C_2_ content and high C_1_ content (Figure 1).

*Levels of TNF*α *and IL-6 induced by PM_10_ and PM_2.5_.* The cytokine responses obtained for the 90 PM_10_ and PM_2.5_ samples varied significantly by season and by size (*p* < 0.05) (Table 2). Samples of both PM_10_ and PM_2.5_ from the rainy-warm season produced a more potent average cell response (TNFα and IL-6) than those from the dry-cold season. TNFα secretion was much higher and more homogeneous than that of IL-6 across different PM exposures (coefficients of variation for TNFα according to season and PM size ranged from 22.5% to 125.4%; coefficients of variation for IL-6 ranged from 59.4% to 243.8%) (Table 2). In both seasons, PM_10_ induced more potent TNFα and IL-6 responses than did PM_2.5_.

**Table 2:** Summary statistics for cytokine production (picograms per milliliter) in response to PM exposures according to PM size and season.

Cytokine^*a*^	PM_10_			PM_2.5_		
	Median	Mean (95% CI)	CV (%)	Median	Mean (95% CI)	CV (%)
TNFα
Dry-cold	60.52*	65.08 (59.03, 71.12)	22.50	5.22*	8.42 (5.23, 11.62)	91.80
Rainy-warm	98.60*	105.63 (79.90, 131.35)	52.02	14.01*	32.24 (13.31, 51.17)	125.40
IL-6
Dry-cold	0.51*	0.62 (0.37, 0.87)	96.93	0.01*	0.22 (–0.001, 0.44)	243.80
Rainy-warm	3.67*	3.89 (2.81, 4.97)	59.41	0.31*	1.01 (0.29, 1.72)	151.20
Abbreviations: CI, confidence interval; CV, coefficient of variation; IL-6, interleukin 6; PM, particulate matter; TNFα, tumor necrosis factor alpha. ^***a***^In picograms per milliliter, after subtracting cytokine levels produced by non-exposed control cells. *Indicates significant differences between seasons by comparing the same cytokine per PM size (*p* < 0.05; Mann–Whitney test).						

**Figure 2:**
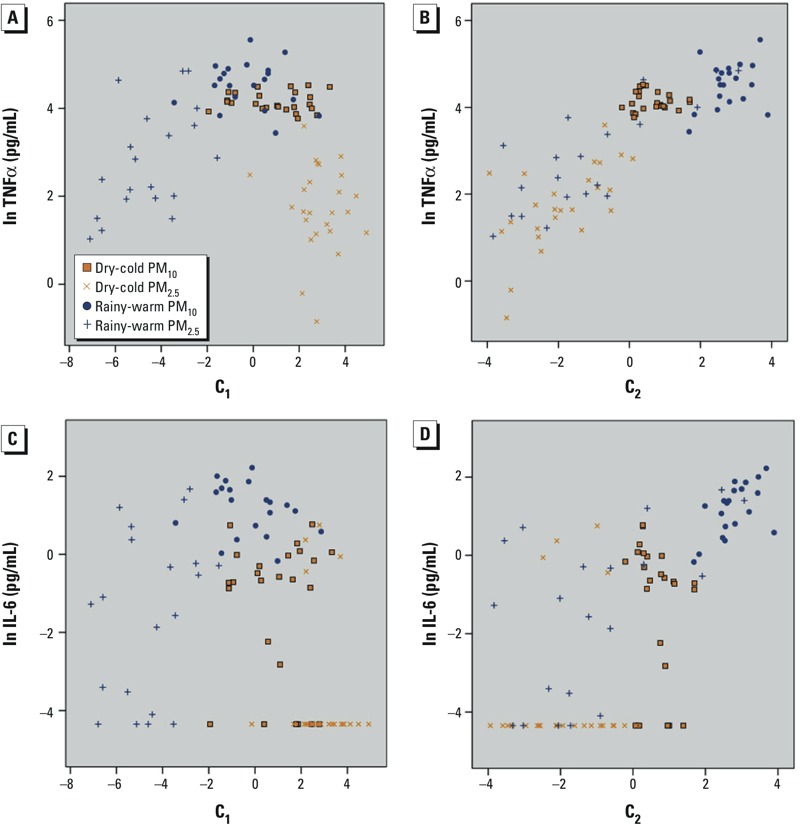
Scatter plots presenting ln(cytokine responses) and PM C_1_ and C_2_ component scores. Patterns of association between TNFα and C_1_ and C_2_ were observed. (*A*) Low levels of PM-induced TNFα secretion occurred with very low and very high values of C_1_, corresponding to PM_2.5_ samples from rainy-warm and dry-cold seasons, respectively. The overall correlation was negative (*r* = –0.32; *p* = 0.002). (*B*) C_2_ and TNFα values showed a significant positive correlation (*r* = 0.65; *p* < 0.001). (*C*,*D*) Highly dispersed low correlation patterns of IL-6 with C_1_ (*r* = –0.36; *p* = 0.000) and C_2_ (*r* = 0.50; *p* = 0.000) were shown. Cytokine values after subtracting cytokine levels produced by non-exposed control cells are presented. The zeroes on the *x*-axis correspond to the mean of all samples, and integer values are based on the observation’s component loading and the standardized value of the original variable, summed over all variables. Abbreviations: C_1_, Component 1; C_2_, component 2; IL-6, Interleukin 6; TNFα, tumor necrosis factor alpha.

Partial correlation coefficients between principal components and ln(cytokine levels) indicated that C_1_ had a statistically significant negative correlation with TNFα (*r* = –0.32; *p* = 0.002) and IL-6 (*r* = –0.36; *p* < 0.001), whereas C_2_ had a statistically significant positive correlation with both cytokines (TNFα: *r* = 0.65; *p* < 0.001; IL-6: *r* = 0.5; *p* < 0.001). However, the C_1_ and TNFα scatter plot showed an inverse U-shape curve, indicating that TNFα secretion in response to PM exposure was highest when C_1_ content was in the middle of the distribution, and TNFα secretion was lowest in response to exposure to PM with very low and very high C_1_ content (Figure 2A). Those extreme values corresponded to PM_2.5_ samples from rainy-warm and dry-cold seasons, respectively. In contrast, the scatter plot and linear regression of C_2_ and TNFα values showed a statistically significant positive correlation (*r* = 0.65; *p* < 0.001) (Figure 2B). Associations of IL-6 with C_1_ and C_2_ were similar to corresponding associations with TNFα; however, the patterns were less clear because the data were more dispersed (Figure 2C,D). C_3_ was not associated with either of the cytokines (*p*-value = 0.407 for TNFα and 0.257 for IL-6), and no further analysis was performed (data not shown).

**Figure 3:**
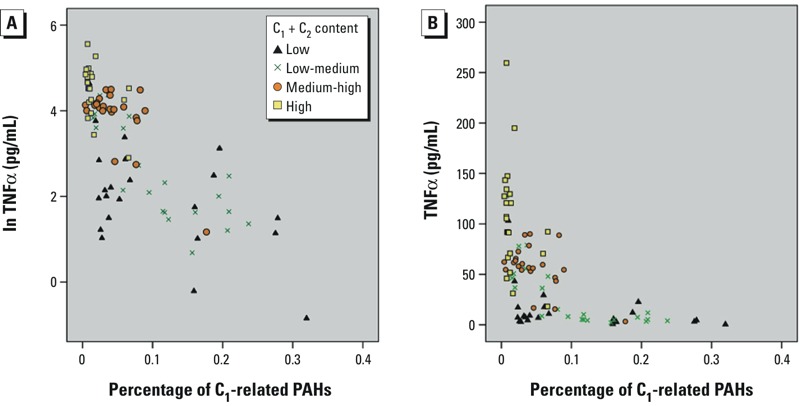
Scatter plot of PM-induced TNFα versus the percentage of PM C_1_-related PAHs. (*A*) The relationship between ln(TNFα) and PM C_1_-related PAHs was negative (adjusted *R* ^2^ = 0.75; probability > *F* = 0.000). (*B*) Untransformed data show that TNFα production is markedly reduced when C_1_-related PAHs > ~ 0.1% or C_1_ + C_2_ content is low. Data points are marked by the category of PM C_1_ + C_2_ content in quartiles. Abbreviations: C_1_, Component 1; C_2_, component 2; PAH, Polycyclic aromatic hydrocarbon; TNFα, tumor necrosis factor alpha.

TNFα was negatively associated with C_1_-related PAHs (Figure 3A,B). Furthermore, based on a regression model adjusted for PM size and C_1_ + C_2_ content, the percentage of PAHs present in C_1_ (C_1_-related PAHs) was negatively associated with ln(TNFα production) in response to PM exposure (regression coefficient *–*8.21; 95% CI: –10.7, –5.7 for the percentage of C_1_-related PAHs) (see Supplemental Material, Table S5). In addition, PM size (PM_10_ > PM_2.5_) and C_1_ + C_2_ content (concentration dependent) were significant positive predictors of TNFα production in response to PM exposure. When the proportion of C_1_-related PAHs was > 0.1%, PM-induced TNFα levels were low regardless of the C_1_ + C_2_ content; this phenomenon is more clearly seen with untransformed TNFα values (Figure 3B). For IL-6, the results were not statistically significant (data not shown), likely because of data dispersion and the number of observations with very low IL-6 concentration values (Figure 2C).

## Discussion

We have described the inflammatory effects of PM obtained from five sites in Mexico City during the rainy-warm and dry-cold seasons, and we have demonstrated that seasonal variability in PM composition is strongly correlated with its potential to induce exposed cells to secrete TNFα and IL-6. Although other studies have identified PM-related effects linked to season-related changes in composition (Bell et al. 2007; Cheung et al. 2011; Toscano et al. 2011; Traversi et al. 2010), our large number of samples allowed us to identify season-related changes in PM composition that explained the variation in the observed cellular responses.

Epidemiological studies have reported associations between seasonal increases in PM concentrations and adverse health outcomes, including mortality (Moolgavkar 2003; Smith et al. 2000). Increased PM levels are not the only determinant of toxic potential; undoubtedly, chemical composition (Dergham et al. 2012; Totlandsdal et al. 2014) and seasonal changes in chemical composition play fundamental roles (Laden et al. 2000) in determining the toxicity of PM. Other epidemiological (Son et al. 2012) and experimental studies have provided evidence of seasonal biological effects on PM chemical composition (Becker et al. 2005; Camatini et al. 2012; Perrone et al. 2010). Specifically, a greater reduction in cell viability was observed after exposure to PM collected from two Italian cities in the summer versus the winter; summer PM samples had a higher content of sulfates, Al, arsenic (As), Cr, Cu, and Zn (Perrone et al. 2010). Similarly, another Italian study showed greater cell toxicity and a greater pro-inflammatory response after exposure to summer PM_10_ samples, which contained high levels of mineral dust elements, Fe, and endotoxins, versus winter PM_10_ samples, which had lower contents of the same constituents (Camatini et al. 2012).

We also observed seasonal differences in the chemical composition of PM_10_ and PM_2.5_. Concentrations of the anthropogenic-related component (C_1_, rich in V and PAHs) were higher in the winter PM samples (dry-cold season) than in the summer PM samples (rainy-warm season) (PM_2.5_ > PM_10_). A second identified component (C_2_) containing endotoxins and soil elements (e.g., Si, Al) showed opposite seasonal differences (higher in the summer than in the winter), mainly for PM_10_.

Elevated PAH content in PM during the dry-cold season has been observed in previous studies performed in Mexico City (Mugica et al. 2010; Vega et al. 2011). However, no previous studies have described seasonal differences in PM-related PAH content in Mexico City. Studies in other cities have documented higher levels of PAHs during the coldest season of that region (Perrone et al. 2010). Existing studies describing other season-related changes in the chemical composition of PM from Mexico City are limited to the winter and the early spring (Vega et al. 2011).

Seasonal differences in chemical composition are likely related to meteorological conditions (e.g., gas-partition ratios driven by temperature and humidity, windblown dust, thermal inversions, atmospheric mixing layer depth), and to changes in human activities (e.g., traffic patterns) (Mugica et al. 2010; Pandolfi et al. 2014; Vega et al. 2011). Additionally, experimental evidence indicates that ozone co-existing in the atmosphere with wood smoke particles can decrease the PAH content and bioreactivity of the particles (Nordin et al. 2015). During the study period, we observed that during the rainy-warm season, higher levels of ozone were negatively correlated with PM-related PAH content (data not shown). However, further research assessing the impact of atmospheric oxidative reactions on PM-related seasonal toxicity is required.

The pro-inflammatory potential of PM samples showed seasonal variation. Samples from the rainy-warm season were more potent in inducing an inflammatory response than samples from the dry-cold season, and PM_10_ was more potent than PM_2.5_. Pro-inflammatory cellular responses were positively correlated with the component that was rich in soil constituents (C_2_). Other authors have described relationships between the pro-inflammatory potential of PM and its content of endotoxins, Fe, and Cu in samples collected in Europe (Guastadisegni et al. 2010) and its content of endotoxins and carbon in samples collected in the Netherlands (Steenhof et al. 2011). However, neither of those two studies analyzed seasonal variability.

The component C_1_ had a non-linear association with TNFα levels. The TNFα response showed an inverse U-shaped dose–response curve for C_1_. Samples with low and high C_1_ content elicited low levels of TNFα secretion, whereas PM with intermediate C_1_ content elicited high levels of TNFα secretion. High C_1_ content mainly occurred in PM_2.5_, specifically PM_2.5_ collected in the dry-cold season.

Seasonal patterns in cell responses related to the PAH content of PM have been observed. Aung et al. (2011) noted that PM rich in carbon, sulfates, and Cu that was collected in California during the summer stimulated increased expression of genes related to inflammation, whereas PM collected in the winter that had a higher PAH content elicited a reduced inflammatory gene response. Similarly, Camatini et al. (2012) reported that PM collected in Italy during the winter had 10 times more PAHs and induced a lower pro-inflammatory cell response than did PM samples collected in the summer. However, the authors did not describe any effects related to interactions among the PM components or to their PM fractions as we have done in the present paper.

Our analyses indicated that PM-induced TNFα production decreased significantly with increasing PAH content in C_1_, after adjusting for PM size and C_1_ + C_2_ content. When the amount of PAHs in C_1_ reached ≥ 0.1% of the C_1_ + C_2_ content, PM-induced TNFα secretion decreased. To our knowledge, this is the first report describing the relative participation of groups of PM constituents in PM of different sizes and the season-related cellular induction of TNFα secretion using real-world particles. The higher content of soil components in the rainy-warm season PM_10_ was associated with greater cytokine production, whereas the higher content of PAHs in dry-cold season samples of PM_2.5_ and PM_10_ was associated with reduced TNFα production. The higher content of PAHs in PM_2.5_ was linked to a pattern of lower cytokine production when compared with PM_10_.

Other reports support the idea that particle-mediated cell effects are related to interactions of particle constituents that alter physicochemical properties of PM. The reduced bioavailability of PAHs adsorbed to particles is one proposed mechanism for affecting stimulation of cytokine production (Goulaouic et al. 2008). Other researchers observed that *in vitro* PAH-induced cell DNA damage signaling was more potent when PAHs were added in mixtures than when they were tested individually, and responses to mixtures containing high-molecular-weight PAHs were more persistent (Jarvis et al. 2013). Studies in zebrafish indicated that real-world PM rich in PAHs increased embryotoxicity and dioxin-like activity more than samples rich in mineral content (Mesquita et al. 2014). All of these results are consistent with the PAH- and soil-related responses to PM observed in the present study.

Our data suggest that the mechanisms mediating TNFα and IL-6 production after PM stimulation are different. IL-6 production was lower and more dispersed than that of TNFα; additionally, IL-6 had a weaker linear correlation with soil content and did not show a clear association pattern with PAH content. These features suggest the existence of complex interactions between PM components to regulate the induction of IL-6 production, as well as modulatory interactions between TNFα and IL-6, as suggested by Raspé et al. (2013). These authors observed that exposure to various combinations of immunomodulatory amino acids altered TNFα or IL-6 expression after stimulation of monocytes with LPS. Others have described differential interleukin response patterns when testing mixtures of PAHs adsorbed to particles on monocytic cells (Goulaouic et al. 2008).

Future studies are needed to fully understand how PM composition differentially affects signaling and cellular pathways, leading to different health outcomes related to seasonal PM composition. A comprehensive evaluation of PM constituents and cellular responses would certainly increase our understanding of the mechanism involved (Dergham et al. 2012; Totlandsdal et al. 2014). The present study centers on the complexity of PM composition. Thus far, we have explored two main groups/components of chemicals commonly identified in PM samples but have not attempted to isolate specific constituents (e.g., Na, endotoxins) or interactions of constituents within each component. We continue to lack knowledge of specific biological effects attributable to PM constituents; for example, information is limited regarding the simultaneous effects of PM-related PAHs and various cell outcomes such as inflammation, biotransformation, and DNA damage potential (Ovrevik et al. 2010; Teixeira et al. 2012). As previously postulated, PM-induced cell responses result from complex interactions among constituents of the PM mixture (Osornio-Vargas et al. 2011). The present study provides a foundation for future mechanistic studies wherein larger sets of biological effects and PM constituents could be considered. Understanding the interactions between cells and PM constituents is an important step towards bridging toxicological and epidemiological studies. Our results support the hypothesis that seasonal changes in PM composition affect biological responses to PM. The epidemiological cohort study that we are presently conducting will further explore the role of seasonality of air pollution exposures during pregnancy and the potential negative impacts on birth outcomes (O’Neill et al. 2013).

## Conclusions

PM showed seasonal variations in composition and *in vitro* pro-inflammatory effects, which were strongly driven by constituents related to soil sources. However, soil-related elements in samples collected during the dry-cold season, which had higher PAH content, did not trigger a pro-inflammatory response. TNFα secretion did not appear to be induced by exposure to PM when the PAH content of the PM was ≥ 0.1%. However, IL-6 production did not fit this pattern, most likely as a result of complex interactions between PM components, PM size, and/or cytokine cross-talk. These interactions should be considered in future toxicological and epidemiological studies linking PM composition to deleterious effects.

## Supplemental Material

(506 KB) PDF
