# Supplementary material for: TNFα and IL-6 Responses to Particulate Matter in Vitro: Variation According to PM Size, Season, and Polycyclic Aromatic Hydrocarbon and Soil Content
Source: Environ Health Perspect. 2015 Sep 15;124(4):406–12. doi: 10.1289/ehp.1409287 (PMC4829995; doi:10.1289/ehp.1409287)
Supplement: (506 KB) PDF [file ehp.1409287.s001.acco.pdf]

Environ Health Perspect
DOI: 10.1289/ehp.1409287

Note to Readers: *EHP* strives to ensure that all journal content is accessible to all readers. However, some figures and Supplemental Material published in *EHP* articles may not conform to 508 standards due to the complexity of the information being presented. If you need assistance accessing journal content, please contact ehp508@niehs.nih.gov. Our staff will work with you to assess and meet your accessibility needs within 3 working days.

Supplemental Material

TNFα and IL-6 Responses to Particulate Matter *in Vitro*: Variation According to PM Size, Season, and Polycyclic Aromatic Hydrocarbon and Soil Content

Natalia Manzano-León, Jesús Serrano-Lomelin, Brisa N. Sánchez, Raúl Quintana-Belmares, Elizabeth Vega, Inés Vázquez-López, Leonora Rojas-Bracho, Maria Tania López-Villegas, Felipe Vadillo-Ortega, Andrea De Vizcaya-Ruiz, Irma Rosas Perez, Marie S. O'Neill, and Alvaro R. Osornio-Vargas

Table of Contents

Table S1. Descriptive statistics for PM_{10} constituents per season, listed in descending order according to the Dry-cold season constituent percentages.

Table S2. Descriptive statistics for $PM_{2.5}$ constituents per season, listed in descending order according to the Dry-cold season constituent percentages.

Table S3. Constituents excluded from the statistical analysis.

Table S4. PCA-Component Matrix.

Table S5. Regression model for TNFα production (ln-transformed) and the percentage of C_1-related PAHs, adjusted by PM-size and $C_1 + C_2$ content (quartiles).

Figure S1. Radial plot of the C_1, C_2 and C_3 component scores, according to PM-size and season. In PM_{10}, the averages were different between seasons ($p<0.05$), whereas in $PM_{2.5}$ exclusively C_1 was different between seasons. (*) Indicates significant differences between seasons by PM-size ($p<0.05$).

Table S1. Descriptive statistics for PM_{10} constituents per season, listed in descending order according to the Dry-cold season constituent percentages.

	Dry-cold season			Rainy-warm season		
**Constituent**[a]	**Median**	**Mean ± SD**	**%**	**Median**	**Mean ± SD**	**%**
Ca*	36,373.7	35,697.4 ± 5,468.0	52.151	89,749.3	93,481.9 ± 30,757.5	74.045
S*	18,009.7	17,754.4 ± 3,828.2	25.938	13,665.7	16,449.3 ± 10,094.9	13.029
K*	5,167.6	6,284.4 ± 2,699.7	9.181	2,081.0	2,579.2 ± 1,173.5	2.043
Na*	3,897.0	3,994.7 ± 1,204.5	5.836	5,710.4	6,853.3 ± 2,817.6	5.428
Mg	1,425.8	1,484.7 ± 310.7	2.169	1,234.0	1,372.0 ± 437.9	1.087
Si*	1,112.7	1,087.6 ± 276.4	1.589	1,731.5	1,930.3 ± 814.5	1.529
Zn	330.1	475.9 ± 378.7	0.695	334.2	538.2 ± 624.5	0.426
Fe*	463.0	452.2 ± 63.2	0.661	585.8	675.4 ± 291.9	0.535
Cu	143.6	283.3 ± 291.5	0.414	174.5	881.7 ± 1,673.3	0.698
V*	186.7	218.6 ± 95.0	0.319	127.5	146.8 ± 78.6	0.116
Al*	116.9	130.0 ± 44.6	0.190	621.4	786.1 ± 407.8	0.623
Ba	123.6	128.4 ± 44.3	0.188	112.9	142.2 ± 103.0	0.113
Mn	123.2	123.7 ± 21.8	0.181	105.8	129.4 ± 71.8	0.103
Sr*	104.9	108.3 ± 19.3	0.158	124.3	135.4 ± 49.6	0.107
Sb*	79.4	82.9 ± 32.9	0.121	26.9	29.1 ± 17.0	0.023
As*	22.8	31.9 ± 20.3	0.047	12.7	15.9 ± 11.2	0.013
Ni*	19.0	22.0 ± 9.0	0.032	12.0	17.3 ± 11.8	0.014
Acenaphthylene	16.6	18.8 ± 16.1	0.027	21.5	22.6 ± 8.4	0.018
*Fluoranthene**	7.3	8.4 ± 6.2	0.012	1.6	1.6 ± 0.7	0.001
Rb*	7.1	7.3 ± 1.2	0.011	3.3	3.8 ± 1.5	0.003
*Benzo(g,h,i)perylene**	6.9	7.5 ± 3.5	0.011	1.7	2.4 ± 1.8	0.002
Mo	6.8	7.2 ± 2.6	0.011	6.4	7.4 ± 4.0	0.006
Cr*	5.3	7.6 ± 5.7	0.011	7.7	10.5 ± 6.7	0.008
Pb*	5.5	6.8 ± 5.3	0.010	13.7	16.8 ± 10.6	0.013
endotoxins	4.6	5.2 ± 1.6	0.008	4.1	6.6 ± 5.4	0.005
Benzo(b)fluoranthene	4.4	5.1 ± 3.0	0.007	3.2	3.6 ± 1.4	0.003
*Pyrene**	0.2	4.4 ± 8.7	0.006	1.9	2.2 ± 1.2	0.002
*Benzo(a)pyrene**	2.9	3.5 ± 2.4	0.005	0.2	0.2 ± 0.1	0.000
Chrysene	2.7	3.3 ± 2.8	0.005	2.2	2.7 ± 1.3	0.002
Li	2.2	2.3 ± 1.8	0.003	1.9	4.4 ± 5.9	0.004
Benzo(a)anthracene	0.9	1.3 ± 1.4	0.002	0.5	0.6 ± 0.4	0.001
Benzo(k)fluoranthene	0.8	1.1 ± 1.1	0.002	1.0	1.2 ± 0.6	0.001
Total		68,450.2 ± 8,281.2	100.000		126,250.1 ± 44,758.3	100.000

[a] ng/mg

* indicates significant differences between seasons by comparing same constituents ($p < 0.05$; Mann-Whitney test).

PAHs constituents in *Italics*.

Table S2. Descriptive statistics for $PM_{2.5}$ constituents per season, listed in descending order according to the Dry-cold season constituent percentages.

	Dry-cold season			Rainy-warm season		
**Constituent** [a]	**Median**	**Mean ± SD**	**%**	**Median**	**Mean ± SD**	**%**
S*	21,073.5	20,610.2 ± 6,127.2	44.226	5,130.4	5,280.8 ± 2,651.0	19.071
Ca	7,616.4	10,481.2 ± 8,164.9	22.491	7,903.7	15,630.3 ± 18,048.4	56.448
K*	6,095.5	8,061.3 ± 5,386.7	17.298	743.1	840.1 ± 500.2	3.034
Na*	3,069.6	2,880.6 ± 1,197.8	6.181	4,269.9	4,273.5 ± 1,157.9	15.434
Zn*	985.3	1,299.2 ± 923.0	2.788	217.0	226.5 ± 114.3	0.818
Mg*	537.8	667.0 ± 463.8	1.431	214.8	318.9 ± 262.2	1.152
Fe*	443.2	461.1 ± 141.1	0.989	113.7	171.7 ± 161.4	0.620
Cu*	329.3	411.2 ± 251.5	0.882	149.7	197.6 ± 148.7	0.714
V*	322.2	335.2 ± 132.1	0.719	43.3	43.9 ± 26.2	0.159
Si	170.0	287.2 ± 255.5	0.616	186.3	341.1 ± 488.1	1.232
Al*	235.3	274.5 ± 161.7	0.589	92.1	183.1 ± 231.1	0.661
Ba*	120.0	186.3 ± 157.2	0.400	24.2	31.6 ± 24.9	0.114
Sb*	143.2	171.3 ± 80.0	0.368	14.4	15.2 ± 7.4	0.055
Mn*	89.8	106.8 ± 61.3	0.229	23.7	33.9 ± 27.9	0.123
Pb*	72.1	99.1 ± 83.8	0.213	12.8	18.2 ± 13.4	0.066
As*	43.0	61.8 ± 46.5	0.133	5.7	8.4 ± 8.5	0.030
Sr*	44.5	57.2 ± 39.4	0.123	10.3	21.9 ± 24.2	0.079
Ni*	42.4	44.8 ± 18.2	0.096	6.5	7.9 ± 5.4	0.029
*Benzo(g,h,i)perylene**	15.7	16.8 ± 7.5	0.036	1.3	1.4 ± 0.9	0.005
Cr*	7.2	12.9 ± 16.4	0.028	4.3	5.6 ± 4.7	0.020
*Benzo(b)fluoranthene**	10.1	11.4 ± 5.3	0.024	1.7	5.0 ± 7.3	0.018
*Fluoranthene**	10.6	10.5 ± 2.7	0.022	0.7	0.9 ± 0.5	0.003
Mo*	9.7	10.3 ± 4.6	0.022	2.0	2.8 ± 2.3	0.010
*Benzo(a)pyrene **	8.1	9.5 ± 4.6	0.020	0.1	0.1 ± 0.0	0.000
Rb*	7.1	7.4 ± 2.2	0.016	0.8	1.5 ± 2.2	0.005
*Acenaphthylene**	0.8	6.1 ± 15.7	0.013	17.6	21.0 ± 9.8	0.076
*Chrysene**	4.8	5.5 ± 2.8	0.012	0.7	0.9 ± 0.6	0.003
*Benzo(k)fluoranthene **	3.6	4.4 ± 2.6	0.009	0.3	0.4 ± 0.3	0.001
*Benzo(a)anthracene**	3.5	3.9 ± 2.1	0.008	0.2	0.2 ± 0.2	0.001
Pyrene	0.3	3.0 ± 5.0	0.007	0.7	0.9 ± 0.7	0.003
endotoxins*	2.1	2.3 ± 1.7	0.005	0.5	1.0 ± 1.2	0.003
Li	2.1	2.3 ± 2.7	0.005	1.4	3.4 ± 4.2	0.012
Total		46,602.4 ± 16,132.5	100.000		27,689.5 ± 21,017.9	100.000

[a] ng/mg

* indicates significant differences between seasons by comparing same constituents ($p < 0.05$; Mann-Whitney test).

PAHs constituents in I*talics*.

Table S3. Constituents excluded from the statistical analysis.

	PM_{10}		$PM_{2.5}$	
	**Dry-cold**	**Rainy-warm**	**Dry-cold**	**Rainy-warm**
**Constituent**[a]	**mean ± SD**	**mean ± SD**	**mean ± SD**	**mean ± SD**
Acenaphthene	2.5 ± 5.1	n/d ± --	1.96 ± 2.6	n/d ± --
Anthracene	3.17 ± 2.81	n/d ± --	4.21 ± 2.98	n/d ± --
Dibenz(a,h)anthracene	n/d ± --	0.72 ± 0.49	0.85 ± 1.13	0.27 ± 0.39
Phenanthrene	n/d ± --	12.05 ± 3.29	9.82 ± 14.14	3.43 ± 3.04
Fluorene	0.89 ± 0.9	n/d ± --	1.87 ± 5.25	n/d ± --
Indeno(1,2,3-cd)pyren	4.44 ± 8.85	n/d ± --	9.74 ± 7.57	n/d ± --
Naftene	6.3 ± 11.42	n/d ± --	9.28 ± 7.14	n/d ± --
Silver(Ag)	n/d ± --	0.23 ± 0.03	n/d ± --	n/d ± --
Berryllium(Be)	n/d ± --	n/d ± --	n/d ± --	n/d ± --
Bismuth(Bi)	n/d ± --	n/d ± --	n/d ± --	n/d ± --
Cadmium(Cd)	4.21 ± 4.68	n/d ± --	16.07 ± 11.63	n/d ± --
Cobalt(Co)	1.49 ± 0.33	1.14 ± 0.94	0.91 ± 0.4	0.7 ± 0.41
Mercury(Hg)	n/d ± --	n/d ± --	n/d ± --	n/d ± --
Scandium(Sc)	n/d ± --	0.23 ± 0.22	n/d ± --	0.13 ± 0.06
Tin(Sn)	27.39 ± 7.49	n/d ± --	58.31 ± 26.4	n/d ± --
Terbium(Tb)	n/d ± --	n/d ± --	n/d ± --	n/d ± --

[a] ng/mg

n/d = below detectable limits. PAHs constituents in *Italics*.

Table S4. PCA-Component Matrix.

Constituent	C_1	C_2	C_3
K	.919		
V	.896		
S	.865		
Benzo(a)pyrene	.849		
Benzo(a)anthracene	.844		
Fluoranthene	.838		
Chrysene	.834		
Benzo(k)fluoranthene	.833		
Benzo(g,h,i)perylene	.786		
Fe	.727		
Zn	.694		
Benzo(b)fluoranthene	.606		
Ca		.935	
Mg		.762	
endotoxins		.719	
Si		.646	
Al		.646	
Na		.637	
Pyrene			.621
Acenaphthylene			.501

Loadings for the three principal components extracted (C_1, C_2, C_3). Values below 0.5 were not shown to highlight the strength of the relationships between constituents and specific principal components. PAHs constituents in *Italics*

Table S5. Regression model for TNFα production (ln-transformed) and the percentage of C_1-related PAHs, adjusted by PM-size and $C_1 + C_2$ content (quartiles).

ln-TNFα	Estimated coefficients	t	P>t	95% CI
C_1-related PAHs (%)	-8.21	-6.68	0.000	(-10.66, -5.77)
PM-size	1.02	4.29	0.000	(0.55, 1.49)
C_1+C_2 mass quartile 2	0.41	1.91	0.059	(-0.02, 0.85)
C_1+C_2 mass quartile 3	0.60	2.16	0.034	(0.05, 1.16)
C_1+C_2 mass quartile 4	0.89	3.01	0.004	(0.30, 1.48)
_const.	2.83	14.54	0	(2.45, 3.22)

n=90; F (5,84)=53.46; Prob>F=0; R-squared=0.76; Adj R-squared=0.7; Root MSE=0.70; C_1+ C_2 mass quartile 1 is the reference for quartile adjustment. According to this model an increase of one-unit in C_1-related PAHs would result in $(e^{-8.21} – 1)*100$ percentage change in TNFα production.

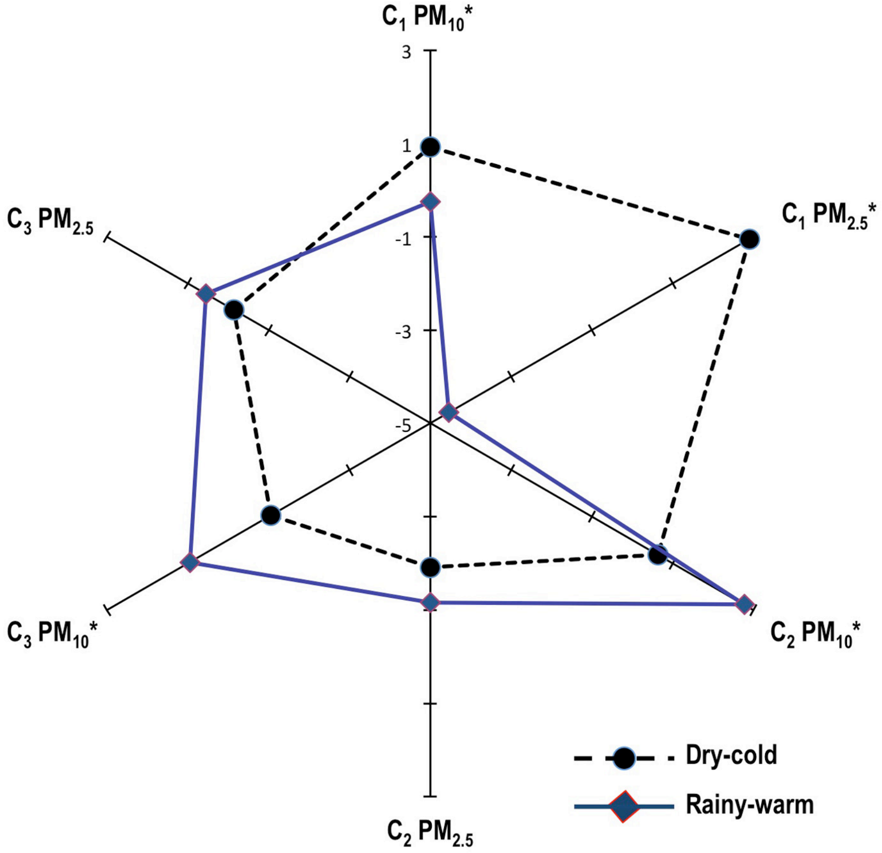

Figure S1. Radial plot of the C_1, C_2 and C_3 component scores, according to PM-size and season. In PM_{10}, the averages were different between seasons ($p<0.05$), whereas in $PM_{2.5}$ exclusively C_1 was different between seasons. (*) Indicates significant differences between seasons by PM-size ($p<0.05$).
